# Protocol for canalostomy in rats to enable inner ear injection

**DOI:** 10.1016/j.xpro.2026.104645

**Published:** 2026-06-20

**Authors:** Lei Zhang, Hanwen Zhou, Wenjie Liu, Siyue Wang, Huiyue Wang, Jinyu Wang, Ning Cong, Jing Wang

**Affiliations:** 1 Department of Otorhinolaryngology, Eye & ENT Hospital of Fudan University, Shanghai 200031, China

**Keywords:** Genetics, Model Organisms, Neuroscience

## Abstract

Here, we present a protocol for precise inner ear access in rats using a refined canalostomy technique. We describe steps for surgical exposure of the mastoid bone, localization of the target semicircular canal, fenestration using a cranial drill, and quantitative microinjection of agents. We further validate this approach by presenting fluorescence-based assessment results. This standardized approach enables reproducible delivery of chemicals or viruses into the inner ear.

## Highlights

• Steps for fabricating the microinjection probe
• Instructions for surgical exposure of the rat mastoid bone
• Guidance on identifying and fenestrating the semicircular canal
• Procedures for volume-controlled inner ear injection

## Abstract (editor highlights)

Publisher’s note: Undertaking any experimental protocol requires adherence to local institutional guidelines for laboratory safety and ethics.

## Graphical abstract

**Figure:**
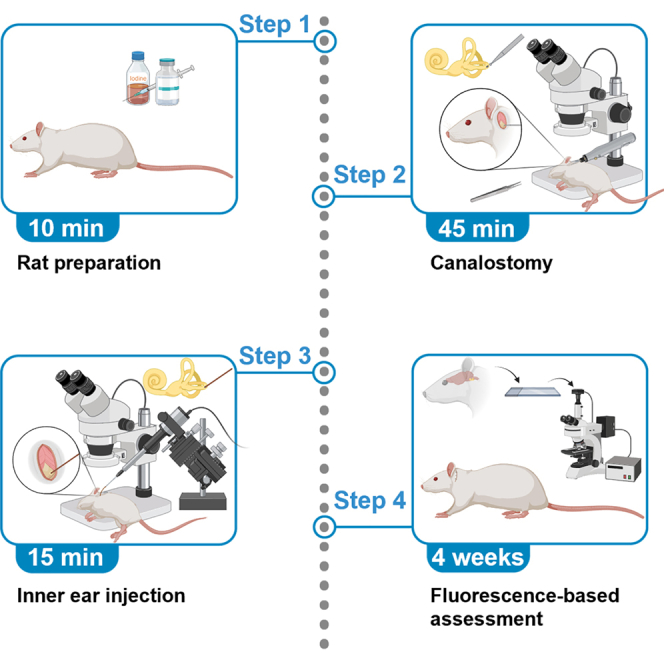

## Teaser

Here, we present a protocol for precise inner ear access in rats using a refined canalostomy technique. We describe steps for surgical exposure of the mastoid bone, localization of the target semicircular canal, fenestration using a cranial drill, and quantitative microinjection of agents. We then detail procedures for validation through fluorescence-based assessment. This standardized approach enables reproducible delivery of chemicals or viruses into the inner ear.

## Before you begin

### Innovation

Injecting substances into the inner ear is essential for auditory and vestibular research. In rats, common surgical routes, such as the posterior tympanotomy via a retroauricular incision or the inferior tympanotomy via a ventral cervical incision,^1^ are used to access the round window membrane or the stapes footplate for injection. These methods are technically challenging due to a narrow operative window and carry risks of damaging critical structures like the ossicular chain or stapedial artery, potentially leading to hearing and vestibular dysfunction. In contrast, canalostomy bypasses the tympanic cavity and critical middle ear structures. It causes minimal auditory damage^2^^,^^3^ and vestibular impairment,^4^^,^^5^ offering a preferable alternative. While the lateral semicircular canal (LSC) and posterior semicircular canal (PSC) in mice form the inferior and posterior borders of the mastoid bone, respectively, and can be fenestrated directly with a needle,^2^ their position in rats is less superficial. In rats, a micro-drill must be used to fenestrate the semicircular canal. We refined existing rat canalostomy techniques^4^ into a standardized, reproducible surgical protocol. Using well-defined anatomical landmarks, our method enables consistent and efficient identification of the mastoid bone. We further detail the procedures for localizing and identifying the semicircular canal during mastoid bone drilling. We also integrate the method for quantitative inner injection in mice^3^^,^^6^ into our protocol. The successful delivery of both DiI and AAV2/retro into the inner ear demonstrates the utility of this approach for experimental applications.

### Institutional permissions

All procedures and animal surgeries were conducted according to the guidelines of the Institutional Animal Care and Use Committee of Fudan University. All efforts were made to minimize the number of animals used and to mitigate the suffering they endured.

**CRITICAL:** Any experiments on live vertebrates or higher invertebrates must be performed in accordance with relevant institutional and national guidelines and regulations. Users are reminded that they will need to acquire the necessary animal permissions from the relevant institutions.

### Preparation of the microinjection probe

**Timing: 45 min**

**Figure 1:**
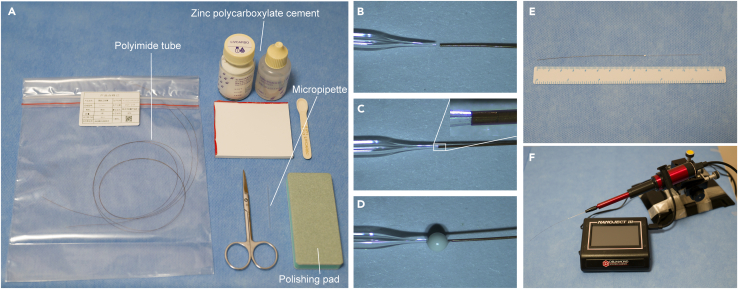
Preparation of the microinjection probe (A) Materials required for preparation of the microinjection probe. (B) Polyimide tube and polished pipette tip. (C) Polyimide tube fitted over the polished tip of the micropipette. (D) Application of a thin layer of zinc polycarboxylate cement to fully encapsulate the joint. (E) Size of the microinjection probe. (F) Nanoject III pump with a sterilized microinjection probe mounted into its capillary holder.

The following steps describe the fabrication of a custom microinjection probe for precise inner ear delivery (Figure 1).

1. Pull glass capillary replacement tubes (compatible with the Nanoject III microinjection pump) into micropipettes using a two-stage vertical pipette puller with the following parameters: Step 1 temperature, 62.8°C; Step 2 temperature, 48.8°C.
2. Polish the pipette tips on a polishing pad. Ensure the outer diameter of the polished tip is smaller than the inner diameter of the polyimide tube (0.1 mm) (Figure 1B).
3. Cut an 8–10 cm segment of polyimide tube.
4. Fit the polyimide tube securely over the polished tip of the micropipette (Figure 1C).
5. Apply a thin layer of zinc polycarboxylate cement to fully encapsulate the joint. Allow the cement to cure completely before proceeding (Figure 1D).
6. Sterilize the assembled probe by autoclaving in a standard sterilization pouch at 121°C for 30 min. After sterilization, store the probe in a small, sealed sterilization tray within a dry, clean environment until ready for use.

### Preparation of the surgical setup

**Timing: 45–60 min**

**Figure 2:**
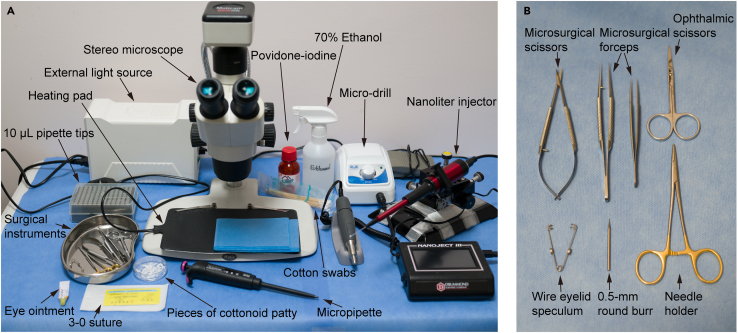
General surgery setup (A) Materials required for canalostomy and subsequent inner ear injection. (B) Surgical instruments.

The following steps detail the preparation of the surgical environment and equipment, ensuring that all sterile and powered components are correctly arranged, tested, and ready for the subsequent surgical procedure (Figure 2).

7. Disinfect the surgery bench, stereo microscope, micro handheld cranial drill and microinjection pump with 70% ethanol.
8. Cover the surgery bench with sterile drapes and secure them in place with tape.
9. Arrange all equipment and supplies on the bench, ensuring they are within easy reach.
10. Cover the heating pad with a sterile drape and absorbent paper, then position it under the microscope.
11. Switch on the warming pad and set it to maintain a target temperature of ∼30°C.
12. Turn on the stereo microscope, external light source, cranial drill and the microinjection pump.
13. Aseptically transfer the entire sterilized instrument tray from its pouch onto the sterile drape.
14. Arrange the instruments in a logical order within easy reach for the procedure.

***Note:*** Before surgery, sterilize all required surgical instruments by autoclaving. Allow the instrument tray to cool completely within its sealed autoclave pouch. For sequential surgeries, to maintain sterility and efficiency between animals, prepare two identical sets of instruments. Sterilize both sets by autoclaving before starting the surgical series. Rotate between the sets for each animal. Immediately after use, immerse the used set in 2% glutaraldehyde solution for at least 20 min for cold sterilization. Subsequently, rinse the set thoroughly with sterile water to remove all chemical residue before its next use in surgery.

15. Mount a sterile 0.5-mm round burr onto the cranial drill.
16. Using sterile scissors, cut a single sterile cottonoid patty into small pieces (∼1 cm in length) and place them in a sterile cell culture dish.
17. Aseptically mount a new, sterilized microinjection probe into the capillary holder of the Nanoject III microinjection pump (Figure 1F).

**CRITICAL:** To prevent cross-contamination and ensure consistent flow, the microinjection probe must be new for each experiment.

***Optional:*** For injecting viscous aqueous solutions, the glass micropipette should be filled with paraffin oil before mounting to increase the driving pressure.

18. Test the probe and its connection to the pump by aspirating and then expelling a small volume of saline to verify patency and confirm a leak-proof seal.

**CRITICAL:** Before any in vivo injection, the integrity of the fluid path must be verified.

### Preparation of the animal

**Timing: 10 min**

19. Weigh the rat and induce surgical-plane anesthesia via intraperitoneal injection of a ketamine-xylazine mixture (80 mg/kg ketamine and 10 mg/kg xylazine). Monitor the depth of anesthesia by loss of pedal reflex.
20. Shave the fur over the retroauricular region using electric clippers. Carefully remove all visible loose hair from the shaved skin by wiping it with a sterile, lint-free gauze pad moistened with sterile saline.
21. Apply chlortetracycline hydrochloride eye ointment to both corneas to prevent desiccation, and additionally, cover the exposed eye with a strip of tape that has a central tissue paper pad to shield it from the surgical light.

## Key resources table

**Table:** 

REAGENT or RESOURCE	SOURCE	IDENTIFIER
**Bacterial and virus strains**		

AAV2/retro-*hSyn*-*EGFP*-*WPRE*-*hGH*	Wuhan Vimicro Corporation	Cat#PT-1990
AAV2/retro-*hSyn*-*mCherry*-*WPRE*-*hGH*	Wuhan Vimicro Corporation	Cat#PT-0100

**Chemicals, peptides, and recombinant proteins**		

1,1′-Dioctadecyl-3,3,3′,3′-tetramethylindocarbocyanine perchlorate (DiI)	Thermo Fisher Scientific	Cat#D3911
2% glutaraldehyde solution	LIRCON	N/A
Paraffin oil	LIRCON	N/A
Ketamine (100 mg/mL)	Fujian Gutian Pharmaceutical Co., Ltd.	N/A
Xylazine (100 mg/mL)	Jilin Huamu Animal Health Products Co., Ltd.	N/A
Chlortetracycline hydrochloride eye ointment	Beijing Shuangji Pharmaceutical Co., Ltd.	N/A
Carprofen	MedchemExpress	Cat#HY-B1227
Hydration gel	Generic	Generic

**Experimental models: Organisms/strains**		

*Rattus norvegicus*, strain Sprague-Dawley, male/female, 6 weeks old	Shanghai Jiesijie Laboratory Animal Co., Ltd.	N/A

**Other**		

Microinjection pump (Nanoject III)	Drummond Scientific	Cat#3-000-207
Glass capillary replacement tubes	Drummond Scientific	Cat#3-000-203-G/X
Two-stage vertical pipette puller (Narishige PC-100)	Narishige International	N/A
Polishing pad, 1000 grit	Generic	Generic
Micro handheld cranial drill	RWD Life Science	N/A
Polyimide tube (inner diameter: 0.1 mm)	Shanghai Qijie Polymer Material Co., Ltd.	N/A
Vetbond™ tissue adhesive	3M	Cat#1469SB
Zinc polycarboxylate cement (LIVCARBO™ Luting Carboxylate Cement)	Erzhichina Dental	Cat#C000132-1
Tape	3M	Cat#1527C-0
Heating pad	Generic	Generic
Stereo microscope	Zeiss	Stemi 305
0.5-mm round burr	RWD Life Science	Cat#78040
Cottonoid patty	Codman	Cat#80-1404
Electric clippers	Generic	Generic
Ophthalmic scissors (10 cm, straight/sharp tips)	JZ CLASSIC	Cat#Y00030
Microsurgical scissors	JZ CLASSIC	Cat#WA1030
Wire eyelid speculum (3 × 5 mm blades, 5 mm depth, maximum spread: 15 mm)	Suqian Wenchuang Medical Device Co., Ltd.	Cat#G102B
Microsurgical forceps	JZ CLASSIC	Cat#WA3040
#5 forceps	FST	Cat#11252-20
Suture 3-0	Ethicon	Cat#W9932

## Materials and equipment

**Table:** Ketamine-xylazine mixture

Reagent	Final concentration	Amount
Ketamine (100 mg/mL)	8.0 mg/mL	4.0 mL
Xylazine (100 mg/mL)	1.0 mg/mL	0.5 mL
0.9% (w/v) NaCl solution	N/A	45.5 mL
**Total**	**N/A**	**50.0 mL**

Store at 4°C in the dark for up to 1 month.

***Alternatives:*** This protocol employs the Nanoject III microinjection pump for precise fluid delivery. Conventional syringe pumps, which are more accessible and economical, can serve as alternatives. Their primary advantages are substantially lower cost and the capacity to handle volumes exceeding the Nanoject III’s 4 μL limit. However, their use necessitates a custom assembly, typically configured in two ways: either a microsyringe connected directly to series-connected capillaries of different diameters, or a microsyringe connected to a glass micropipette that is then fitted with a capillary at its tip. These assemblies often leak more easily, exhibit reduced volumetric accuracy, and have a larger dead volume, leading to increased reagent waste. When considering alternatives for retraction, the wire eyelid speculum from World Precision Instruments (5 × 5 mm blades, 5 mm depth, maximum spread: 15 mm) offers a practical alternative.

## Step-by-step method details

This section provides detailed instructions for: (a) exposing the mastoid bone and fenestrating the LSC or PSC (canalostomy), (b) inner ear injection through the semicircular canal aperture (Methods Video S1), and (c) postoperative care following surgery.

Methods Video S1. Surgical procedure for canalostomy and inner ear injection in rats, related to steps 3–12

### Operative procedure for canalostomy

**Timing: 30–45 min**

**Figure 3:**
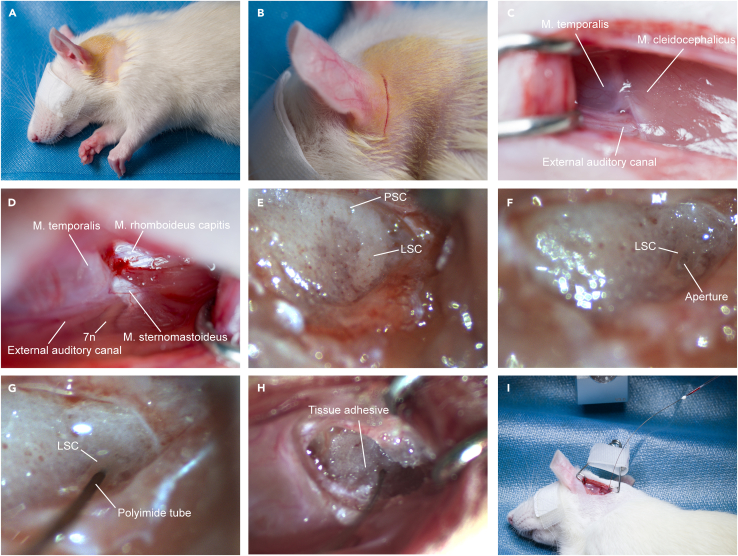
Canalostomy and inner ear injection through the semicircular canal aperture (A) Anesthetized rat in lateral recumbency on a heating pad; surgical site sterilized with povidone-iodine. (B) A 12–13 mm incision made with ophthalmic scissors, perpendicular to the two vascular bundles on the dorsal aspect of the auricle and 1–2 mm posterior to the retroauricular sulcus. (C) The intersection point of the temporalis muscle, external auditory canal and cleidocephalicus muscle. (D) The white, transversely oriented tendon of the rhomboideus capitis muscle. The sternomastoideus tendon lies inferior to it, with the facial nerve located further inferiorly. (E) The surface of the mastoid bone, with LSC at its inferior border and PSC at its posterior border. (F) Fenestration of LSC. (G) Insertion of the polyimide tube into the lumen of LSC. (H) Sealing of the tube with tissue adhesive. (I) Injection of the solution.

Here we describe how to perform surgery to expose the mastoid bone and fenestrate the LSC or PSC. Upon completion of the following steps, the semicircular canal fenestration will be prepared for inner ear injection (Figures 3A–3F) (Methods Video S1 at 0:00–7:03).

1. Position the anesthetized rat in lateral recumbency on the heating pad.
2. Sterilize the surgical site by swabbing the skin three times with povidone-iodine (Figure 3A).
3. Retract the auricle anteriorly and laterally. Make a 12–13 mm incision with ophthalmic scissors, perpendicular to the two vascular bundles on the dorsal aspect of the auricle and 1–2 mm posterior to the retroauricular sulcus (Figure 3B) (troubleshooting 1).
4. Identify the intersection point of the temporalis muscle, external auditory canal and cleidocephalicus muscle (Figure 3C) (Methods Video S1 at 0:42–1:13).
  a. Using microsurgical scissors, dissect deeply in the direction of the mastoid bone through the fascia and adipose tissue until the underlying muscle is fully exposed.
  b. Maintain exposure with a wire eyelid speculum.

***Note:*** If skin tension is noted or if the surgical field visualization is inadequate after eyelid speculum placement, gently extend the incision along its axis as needed using ophthalmic scissors. The main trunk of the facial nerve can be observed inferior to the intersection point of the temporalis muscle, external auditory canal and cleidocephalicus muscle. Superior to the nerve lies the sternomastoideus tendon, which is covered by the cleidocephalicus muscle.

5. Expose the white, transversely oriented tendon of the rhomboideus capitis muscle (Figure 3D) (Methods Video S1 at 1:14–1:56).
  a. Position the tip of microsurgical forceps posterior and superior to the identified intersection, exactly above the sternomastoideus tendon, to bluntly separate the cleidocephalicus muscle perpendicular to its fibers.
  b. Use microsurgical scissors to make a small cut, partially releasing the cleidocephalicus muscle’s anterior attachment.
  c. Secure the separated muscle flap with the wire eyelid speculum to maintain the newly created surgical window.

***Note:*** Using the sternomastoideus tendon (inferior to the rhomboideus capitis tendon) as the inferior boundary, inferior to which lies the facial nerve. Perform all subsequent steps exclusively in the superior region.

6. Expose the surface of the mastoid bone (Figure 3E) (Methods Video S1 at 1:57–2:36).
  a. Make an incision along the tendon insertion site, staying as close as possible to the anterior border of the mastoid bone.
  b. Through this incision, create a small opening in the periosteum.
  c. Starting from this entry point, carefully dissect and elevate the periosteum along the anterior border.
  d. Continue the subperiosteal dissection along the inferior border, elevating the periosteum and reflecting the overlying muscles and tendons that insert onto the bone as part of the continuous flap.
  e. Secure the reflected flap with the wire eyelid speculum to maintain full exposure of the mastoid bone surface.

**CRITICAL:** Keep all incisions as close to the bony borders as possible. This minimizes bleeding and prevents soft-tissue interference, ensuring a clean and accessible bony surface for the subsequent procedure. Do not extend the anterior periosteal incision too superiorly, to avoid the neurovascular structures within the mastoid foramen.

7. Identify and fenestrate the target semicircular canal (Figure 3F) (Methods Video S1 at 2:37–7:03).
  a. Use pieces of cottonoid patty to absorb fluids and maintain a clear surgical field.
  b. Fenestrate the target canal using a 0.5-mm round burr until clear perilymph flows out (troubleshooting 2).
    i. If the canal is directly visible, carefully fenestrate it.
    ii. If the canal is not initially visible, initiate careful drilling in the area adjacent to the inferior border (for LSC) or posterior border (for PSC) of the mastoid bone to locate it. Once located, proceed with fenestration (troubleshooting 3 and 4).
  c. Continue the fenestration until the aperture is sufficiently large to accommodate the insertion of the polyimide tube.

**CRITICAL:** To facilitate canal localization, younger rats (e.g., 200–350 g) are recommended, as their semicircular canals are more superficial and thus more likely to be directly visible. The target semicircular canal is distinguished by its dense, white, avascular bone. In contrast, be aware of intramastoid cavities—superficial, vascular spaces within the mastoid bone that are more shallow than the canals and most commonly overlie the anterior LSC or superior PSC. Drilling into these cavities will induce persistent bleeding. Strictly confine all drilling to the immediate vicinity of the mastoid bone border to avoid accidental perforation into the subarcuate fossa, which risks severe bleeding, exposure of the cerebellar paraflocculus and flocculus, and cerebrospinal fluid (CSF) leakage. During drilling, the appearance of a blue line indicates proximity to the canal lumen. Correct fenestration is confirmed by visual identification of the white endosteum and, upon its puncture, the subsequent outflow of clear perilymph. Should persistent bleeding occur, immediately pause to assess its source. If it originates from an intramastoid cavity, control bleeding with pieces of cottonoid patty and, if feasible, adjust the drilling position slightly; if the cavity directly overlies the target canal, maintain hemostasis and continue drilling at the same location with extreme caution. If subarcuate fossa entry is suspected, cease drilling immediately and relocate the site entirely. To ensure a precise opening, limit bone removal strictly within the identified bony walls of the semicircular canal.

### Operative procedure for inner ear injection through the semicircular canal aperture

**Timing: 15 min**

This section describes the complete inner ear injection procedure following canalostomy. It covers insertion of the polyimide tube into the semicircular canal lumen, sealing the interface with tissue adhesive under careful fluid management, and delivering a controlled volume of solution with a microinjection pump. The section concludes with post-injection steps including pressure equilibration, tube transection, wound closure, and local antisepsis (Figures 3G–3I) (Methods Video S1 at 7:04–10:10).

8. Insert the polyimide tube into the semicircular canal (Figure 3G) (troubleshooting 5) (Methods Video S1 at 7:04–8:04).
  a. Position the rat and the injection device to align the polyimide tube with the axis of the fenestrated semicircular canal.
  b. Use small pieces of cottonoid patty to absorb perilymph from the surgical field, ensuring clear visualization of the aperture.
  c. Gently advance the polyimide tube through the aperture and into the lumen of the semicircular canal by ∼1 mm.
9. Seal the tube with tissue adhesive (Figure 3H) (Methods Video S1 at 8:05–9:18).
  a. Thoroughly absorb all fluid from the surgical field around the polyimide tube using cottonoid patty pieces to ensure a completely dry interface.
  b. Apply 0.5 μL of tissue adhesive with a micropipette around the circumference of the tube.
  c. Wait until the adhesive has polymerized, forming a secure and leak-proof seal.

**CRITICAL:** Upon fenestration and enlargement of the aperture, perilymph flows out rapidly. This flow will gradually slow but not cease. Even after tube insertion, a persistent seepage continues around the tube. The sealing surface must be nearly dry. Excess residual fluid will compromise the tissue adhesive bond, creating gaps that cause injected agents to leak. Therefore, it is imperative to absorb as much fluid as possible and then apply the tissue adhesive promptly to achieve a functional, leak-proof closure before further fluid can accumulate.

10. Initiate the pump to inject the solution at a rate of 1 μL/min for a total volume of 3–4 μL (Figure 3I) (troubleshooting 6 and 7).

***Note:*** Closely monitor the fluid meniscus in the pipette to confirm a steady, visible flow and simultaneously observe the injection site for any signs of solution leakage. The injection rate and volume should be optimized according to the agent used.

11. After the injection is complete, wait for 3 min to allow pressure equalization between the injection device and the inner ear.

***Note:*** Premature transecting of the tube risks reflux of the injected solution from the bone-side cut end of the tube.

12. Transect the polyimide tube with microsurgical scissors, cutting as close to the bone surface as practicable, and immediately seal the bone-side cut end with tissue adhesive (Methods Video S1 at 9:47–10:10).

***Note:*** Minimizing the length of the residual tube stump reduces the risk of post-operative fibrosis, inflammation, or mechanical interference with surrounding tissues. Avoid excessive manipulation of the residual stump, as this may compromise the adhesive seal and cause leakage.

13. Reposition the separated muscles and subcutaneous tissues, then suture the skin incision with 3-0 suture.
14. Disinfect the incision area with povidone iodine; then apply chlortetracycline hydrochloride eye ointment to the incision to prevent postoperative infection.

### Post-operative care

**Timing: 30–60 min**

15. For postoperative analgesia, administer carprofen (5 mg/kg, SC) immediately following wound closure. Repeat the injection at 24-hour intervals for a total of 2–3 days.

***Note:*** When handling the rat for subcutaneous injection, take care to avoid pressure on or contact with the fresh retroauricular incision site to prevent trauma or disruption of the wound.

16. Return the rat to a clean, separate cage in a temperature-controlled recovery area maintained at 25–27°C.

***Note:*** To ensure adequate nutrition and hydration with restricted neck movement following surgery, place both standard food and hydration gel within easy reach inside the cage.

17. Monitor the animal closely until it has fully recovered from anesthesia.
18. Monitor the animal daily for wound infection (e.g., redness, swelling, discharge) for 7 days postoperatively.

***Note:*** If any signs of infection are observed, consult the institutional veterinarian immediately for evaluation and potential systemic antibiotic therapy.

## Expected outcomes

**Figure 4:**
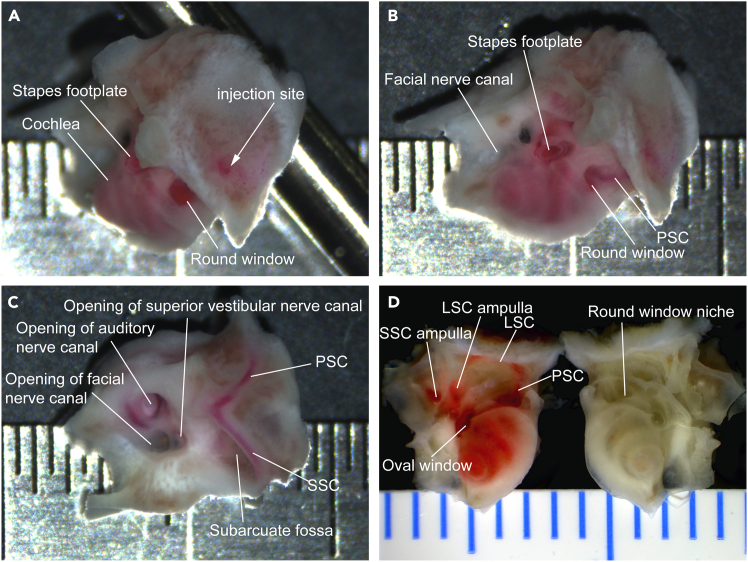
Stereo microscope images of the inner ears of rats injected with DiI following canalostomy Samples were collected 9 days after surgery. (A and B) The extracranial surface. (C) The intracranial surface. (A–C) DiI was injected through the lateral semicircular canal. (D) The extracranial surface, with the injection site on the left and the non-injection site on the right. DiI was injected through the posterior semicircular canal. DiI distributes throughout the semicircular canals, vestibule, and cochlea. Each division on the ruler is 0.5 mm (A–C). Each division on the ruler is 1 mm (D). PSC, posterior semicircular canal. SSC, superior semicircular canal. LSC, lateral semicircular canal.

DiI (0.4 mg/mL in ethanol) was injected into the LSC or PSC to assess its final distribution within the inner ear. By day 9, the dye had spread throughout the semicircular canals, vestibule, and cochlea (Figure 4).

**Figure 5:**
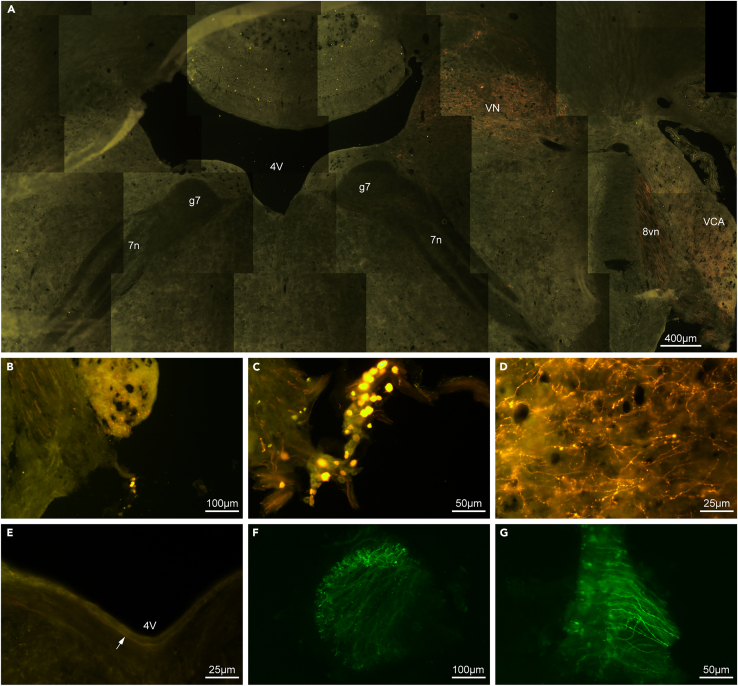
Representative fluorescence microscope images of rat brain stem and inner ear sensory epithelium after canalostomy-mediated AAV injection Tissues were collected 40 days after AAV2/retro-mCherry injection and 285 days after AAV2/retro-EGFP injection. (A) VN, VCA, and 8vn on the injection side expressed mCherry, while no significant expression was observed on the non-injection side (this image is a merged set of tiled images). (B) mCherry expression was observed in the VCA and 8vn, as well as in neurons of the vestibular ganglion. (C) Vestibular ganglion neurons expressed mCherry. (D) mCherry expression was observed in the VN. (E) Some mCherry-expressing nerve fibers cross the floor of the 4V. (F) Afferents of the utricle expressed EGFP. (G) Afferents of the ampulla expressed EGFP. VN, vestibular nuclei. VCA, ventral cochlear nucleus, anterior part. 8vn, vestibular root of the vestibulocochlear nerve. 4V, 4th ventricle. G7, genu of the facial nerve. 7n, facial nerve. Arrow indicates mCherry-positive nerve fibers.

To evaluate the efficacy of canalostomy for inner ear injections, AAV2/retro-mCherry and AAV2/retro-EGFP were injected into the inner ears. Peripherally, fluorescent protein expression was observed in the neural epithelium of the semicircular canals and utricle and the vestibular ganglion (VG). Centrally, mCherry expression was observed in the vestibular root of the vestibulocochlear nerve (8vn), ventral cochlear nucleus, anterior part (VCA) and vestibular nuclei (VN). Some mCherry-positive fibers were found crossing the floor of the fourth ventricle (4V) (Figure 5).

We posit that the injected agent affects the entire inner ear, including its cells and nerve supply, and that the specific outcome depends on the nature of the agent used.

## Limitations

The canalostomy-based injection method enables quantitative delivery but has inherent spatial limitations. Diffusion of injected agents within the bony and membranous labyrinth is difficult to precisely control or predict, as it is influenced by residual perilymph, infusion pressure, and endogenous fluid dynamics. Consequently, the approach does not allow isolated investigation of the vestibular or auditory system. With larger injection volumes, agents may spread via the cochlear aqueduct into the intracranial space, leading to off-target expression. The procedure causes slight physical and physiological disturbance and leaves an implant at the fenestration site. Therefore, monitoring of auditory and vestibular function is recommended. Finally, although this method provides a valuable preclinical model, its translational potential is limited compared to less invasive clinical routes such as round-window injection, as the latter is performed endoscopically via the ear canal, avoiding the need for mastoid drilling and extensive bone opening.

## Troubleshooting

### Problem 1

The rat experiences excessive bleeding during surgery (Step 3).

### Potential solution

In most cases, bleeding in rats is self-limiting. Minor hemorrhage can typically be controlled by applying gentle pressure with cottonoid patty pieces to the site. This protocol does not require the use of electrocautery. To minimize bleeding, avoid contact with visible vessels during dissection where possible; however, note that some vessels are anatomically unavoidable. Additionally, make precise, small increments with the scissors, avoiding taking too large a cut at once. Almost all hemorrhages encountered in this protocol can be managed using these methods.

### Problem 2

The cranial drill bit frequently entangles with or snags the surrounding soft tissue (Step 7b).

### Potential solution

Dissect along the anterior and inferior borders of the mastoid bone; retract the tissue securely with a wire eyelid speculum to optimize exposure; and hold the cranial drill perpendicular to the mastoid bone surface.

### Problem 3

Despite extensive drilling, the horizontal and posterior semicircular canals remain elusive (Step 7bii).

### Potential solution

• Verify the landmark: Ensure that your drilling site is near the posterior or inferior border of the mastoid bone. A common error is insufficient removal of overlying soft tissue, which can obscure the true bony border. Gently push the soft tissue aside with forceps to confirm the actual border.
• Account for anatomical variation: Be aware of significant anatomical variability between rats. The semicircular canals may be located deeper than expected. If initial drilling does not reveal them, continue carefully with deeper bone removal.
• Animal Selection: To reduce technical difficulty, the use of large rats (typically >400 g) is not recommended. In such animals, increased bone density and thickening of the mastoid bone are commonly encountered, substantially impeding the localization of the semicircular canals.

### Problem 4

The intramastoid cavity, a natural anatomical space, is opened during drilling. This leads to persistent oozing into the drill well, obscuring the semicircular canals (Step 7bii).

### Potential solution

Bleeding from the intramastoid cavity is persistent and is readily re-triggered by the mechanical disturbance of drilling shortly after it stops. To manage this, use a cycle of absorbing and drilling: first, absorb the blood with cottonoid patty pieces to clear the view; then, make a few drilling passes in the short time before the bleeding restarts. Repeat this cycle until the wall of the semicircular canal is drilled through. Once opened, the flow of perilymph will wash away the blood, and the bleeding usually stops. To minimize this challenge from the outset, using smaller rats (e.g., 200–350 g) is strongly recommended.

### Problem 5

Insertion of the polyimide tube through the semicircular canal aperture is possible but very difficult (Step 8).

### Potential solution

• Ensure the semicircular canal aperture is sufficiently large.
• Cut the tip of the polyimide tube at a beveled angle.
• Direct the tube toward the non-ampullary side of LSC and the ampullary side of the PSC, respectively, as the anatomical inclination at these directions naturally aligns with the tube’s intended trajectory.

### Problem 6

During the injection process, the fluid meniscus in the pipette fails to descend, descends minimally, or oscillates back and forth (Step 10).

### Potential solution

These observations indicate partial or complete probe obstruction, elevated inner ear pressure, high fluid resistance, or a leak in the system. Preventive measures include using a new probe for each experiment, verifying probe patency and system seal integrity before use, removing precipitates from the injection solution by centrifugation or vortexing, and pre-filling the pipette with paraffin oil for viscous aqueous solutions. While switching the microinjection pump to manual mode allows for a swift increase in internal pressure, thereby causing a rapid descent of the fluid meniscus, this approach leads to uncontrolled injection volume and risks damaging the inner ear. Furthermore, due to the sealing of the tissue adhesive, withdrawing the polyimide tube to replace the probe and reattempt injection is not recommended.

### Problem 7

The solution leaks from the edges of the tissue adhesive during injection (Step 10).

### Potential solution

This issue can be prevented by thoroughly clearing soft tissue from the edges of the mastoid bone, completely drying the bone surface around the tube, and applying 0.5–1 μL of additional tissue adhesive if leakage is anticipated. Perform the injection in multiple aliquots. In the event of leakage during the initial aliquot, absorb the escaped solution, reinforce the seal at the leak site with tissue adhesive, and note the volume of leakage, and then proceed with the next aliquot.

## Resource availability

### Lead contact

Further information and requests for resources and reagents should be directed to and will be fulfilled by the lead contact, Jing Wang (wangjing6437@163.com).

### Technical contact

Technical questions on executing this protocol should be directed to and will be answered by the technical contact, Lei Zhang (lei.zhang.research@gmail.com).

### Materials availability

This study did not generate new unique reagents.

### Data and code availability

No datasets or code were generated or analyzed during this study.

## Acknowledgments

We thank Shijie Liu (Master’s student, Eye & ENT Hospital of Fudan University, 2019–2022) for his preliminary anatomical exploration and Dr. Juanmei Yang (Eye & ENT Hospital, Fudan University) for providing access to the Nanoject III microinjection pump. This work was supported by grants from the National Key Research Program Project (2024YFC2418302) and the 10.13039/501100001809National Natural Science Foundation of China (81870724). The graphical abstract was created with BioRender.com.

## Author contributions

L.Z. conceived and designed the study protocol, led the anatomical exploration and technical optimization, and wrote the manuscript. H.Z. and W.L. co-optimized the methodology and assisted with experiments and manuscript revision. S.W. and H.W. assisted with experiments. Jinyu Wang and N.C. provided technical support and manuscript feedback. Jing Wang supervised the project and secured funding. All authors approved the final manuscript.

## Declaration of interests

The authors declare no competing interests.
